# Effective chair training methods for neuroscience research involving rhesus macaques (*Macaca mulatta*)

**DOI:** 10.1016/j.jneumeth.2019.02.001

**Published:** 2019-04-01

**Authors:** Stuart Mason, Elsie Premereur, Vassilis Pelekanos, Andrew Emberton, Paul Honess, Anna S Mitchell

**Affiliations:** a Department of Experimental Psychology, University of Oxford, Oxford, OX1 3SR, United Kingdom; b Laboratory for Neuro- and Psychophysiology, KU Leuven, Leuven, Belgium; c Biomedical Services, University of Oxford, Oxford, United Kingdom; d Animal Welfare and Behaviour Consultant, United Kingdom

**Keywords:** Non-human primate, *Macaca mulatta*, Welfare, Positive reinforcement training, Food control protocol, Neuroscience, Primate chair, Electrophysiology, Behaviour, Cognition, Conditioning

## Abstract

**Background:**

Neuroscience research using macaques remains fundamental in our endeavours to understand how the human brain functions. Applying the refinement principle of the 3Rs is essential to optimise the monkeys’ welfare and still produces high quality science.

**New method:**

Here we document refinements in our training methods for acclimation to transport devices (i.e. primate chair or transport box) while working with 46 male rhesus macaques. Our training methods always used positive reinforcement training (PRT). However, PRT was sometimes combined with negative reinforcement training (NRT), but not pole and collar techniques, to successfully transfer each monkey from its home enclosure to its transport device.

**Results and comparison with existing methods:**

Training monkeys in pairs or groups, and starting their PRT training upon arrival within the unit reduced the days required to acclimate them. While the use of PRT is essential to establish a positive relationship with monkeys, NRT techniques are sometimes necessary, and are most effective when withdrawn immediately once the monkey makes the desired response, to reduce the days of acclimation. Once acclimatised to their chair, monkeys succeeded within 10 days to present their head voluntarily for neck-plating using PRT. Space reducers inside the chairs also facilitated head presentations for some monkeys.

**Conclusions:**

Acclimating (shaping) the monkeys to transport devices can be a stressful experience for monkeys and trainers. The adaptations to our training substantially *reduced* the days spent on this stage. We view this reduction in days as an effective implementation of the 3Rs (refinement) in monkey neuroscience research.

## Highlights

• Refining training for monkeys in neuroscience is essential to optimise their welfare.
• Refinements still produce high quality science.
• Pair- or group-training monkeys helps acclimate them quicker to transport devices.
• Commencing positive reinforcement training on arrival facilitates acclimation.
• Negative reinforcement techniques used effectively are also sometimes necessary.

## 1 Introduction

Rhesus macaques (*Macaca mulatta*) remain an essential and valuable resource in fundamental neuroscience research (Phillips et al., 2014; Roelfsema and Treue, 2014; SCHEER, 2017; Mitchell et al., 2018). This research typically involves monkeys undergoing testing in cognitive and behavioural tasks while performance assessments and physiological parameters are measured, and electrophysiological recordings of brain cells (neurons) or neuroimaging is conducted. To participate in these experiments that further our fundamental knowledge about higher brain functioning, monkeys are typically trained to enter primate chairs or transport boxes (t-box) from their home enclosures. Next, the animals are wheeled to experimental set-ups, where they learn to perform tasks, and are acclimated to the neuroscience techniques required to collect critical data. The key to collecting these data from each monkey over its study duration is to ensure that the monkey continues to cooperate and has optimal welfare standards, which produces good science. Consequently, those working with non-human primates must support the animal’s welfare, implement the 3Rs (Russell and Burch, 1959), adhere to the highest ethical standards, and successfully train monkeys within the time frames of the neuroscience research constraints thus helping to reduce the overall cost to the monkeys.

Most primate units use somewhat standard yet purpose-designed primate chairs. However, the reported timings for successfully training rhesus macaques to acclimate to these transport/restraint devices varies greatly between different primate units (Bliss-Moreau et al., 2013; McMillan et al., 2014, 2017). For example, McMillan et al. (2017) reported the average time required ‘to prepare monkeys for chair restraint was most often 2–8 weeks’. These differences suggest there are variations in the use of training methods, although the temperament of an individual monkey (Capitanio, 2017; Coleman et al., 2015; Coleman, 2012, 2017), and/or the use of pole and collar techniques (Bliss-Moreau et al., 2013; McMillan et al., 2014) are also likely to be mitigating factors.

In a recent survey of training methods for primates, respondents from macaque units across the world reported that they used either positive reinforcement training (PRT), or a combination of PRT and negative reinforcement training (NRT) techniques to transfer the monkeys from their home enclosures to the transport devices, although many additional techniques were documented (see Figure 9: McMillan et al., 2017). PRT, which incorporates well-studied principles in experimental psychology, namely reinforcement learning and operant and Pavlovian conditioning, is a method that is widely used when training many different species of mammals (Mellen and Ellis, 1996). PRT should increase cooperative behavior as it typically involves the use of highly appetitive rewards for desired behavior, and it is also recognised as a refinement and enrichment strategy for non-human primates (Bloomsmith et al., 2018; Laule et al., 2003; Prescott and Buchanan-Smith, 2003; Schapiro et al., 2003).

The initial training (shaping) of monkeys to acclimate to their transport device is potentially a stressful event for both the monkey and their trainer. In neuroscience experiments involving rhesus macaques in Oxford, opportunities for refinements are sought, while maintaining excellent reproducible and reliable science. Thus the goal of the current set of experiments was to refine our own training methods in order to further optimise the initial acclimation to a transport device. During the course of developing these refinements, we have substantially *reduced* the amount of time required for monkeys to reliably enter transport devices and acclimate to initial neck-restraint training compared with previously reported timings. We compared whether training monkeys individually or as part of a group, or pair, was more effective, and whether the use of just PRT, or predominantly PRT, combined with NRT techniques, was a more effective method in reducing the overall time involved in acclimation. The end training goal in all experiments was for the monkeys to acclimate quickly to the transport devices and where applicable, after acclimating to the chair, reliably participate in neck restraint training. We defined the monkeys as being acclimated when they were reliably entering the transport device on 3 consecutive training sessions, and, where applicable for primate chair training, when no NRT was required for reliable neck restraint training. There are several benefits to refining the training methods to reduce the overall time spent acclimating them to the transport devices and beginning the PRT training sooner. These refinements can potentially reduce the total time the monkey needs to spend in an experimental setting (Honess and Wolfensohn, 2010), or allows more data to be collected over a fixed period of time.

## 2 Methods and materials

### 2.1 Subjects

In total, 46 rhesus monkeys (all male, aged between 2.5 and 6 years at the beginning of behavioral training) participated in these training procedures. Subjects were involved in many different behavioral and cognitive neuroscience experiments funded by the UK Medical Research Council and the Wellcome Trust. The different groups of monkeys arrived in the primate unit at University of Oxford at different times over a period of 9 years (2009–2017) and participated in neuroscience experiments under the Home Office Project Licences authorised to Dr AS Mitchell. We trained all monkeys to enter their transport devices voluntarily, as the use of pole and collar methods to transfer primates from their home enclosure into a transport device is not allowed in the United Kingdom. All experimental procedures were performed in compliance with the United Kingdom Animals (Scientific Procedures) Act of 1986. The monkeys were socially housed together in same sex groups of between two and eight monkeys. The housing and husbandry were in compliance with, and regulated by European Union guidelines (EU directive 86/609/EEC; EU Directive 2010/63/EU) for the care and use of laboratory animals.

For this paper, we conducted several experiments but not all monkeys contributed to each experiment. The monkeys were allocated to different groups, depending on when they arrived in the unit. In addition, the training methods detailed in this manuscript were being refined over this period so different groups experienced different training methods (see Table 1 and details below), and consequently we were able to assess the impact of these differences in training methods that were adopted.

**Table 1:** Overview of the Groups of monkeys involved in each different training method.

Group	Type of reinforcement	Pair-, Group-, or Individual Training	Transport device order of training	Primate chair – voluntary head presentation	Primate Chair - Neck Plating
Group 1	PRT&NRT	Individual	T-box	N/A	N/A
Group 2	Only PRT	Individual	T-box	N/A	N/A
Group 3	PRT&NRT	Pair or Group	T-box	N/A	N/A
Group 4	Only PRT	Pair or Group	T-box -> Chair	X	
Group 5	PRT&NRT	Pair or Group	T-box -> Chair		X
Group 6	PRT&NRT	Pair	Chair -> T-box		X

#### 2.1.1 Specific details of the different training methods and Groups

Group 1: n = 22. All monkeys were trained individually to enter the t-boxes using a combination of PRT and NRT (see Table 1). One monkey was excluded from the data analysis as it took many weeks before it was willing to interact with the trainer and the t-box to receive rewards. Eventually, after this monkey started interacting, it was paired with its already t-box acclimated cagemate to enter its own t-box.

Group 2: n = 6. Monkeys were trained individually to enter the t-boxes using PRT only, i.e. no NRT was used (see Table 1). This resulted in only a 50% success rate by 21 days (i.e. this method had already exceeded the time taken to train the monkeys in Group 1), so we used a combination of PRT and NRT to acclimate the rest of monkeys from day 22 onwards. Only the 3 monkeys (M25, M26 and M27) who had been successfully acclimated to the transport devices by 21 days were included in our analyses for the PRT only training group.

Group 3: n = 9. All monkeys were trained in pairs or groups to enter the t-boxes using mainly PRT, NRT was required for 1 out of the 9 monkeys (M29: see Table 1). As a consequence of the improvements observed from Group 3, all monkeys in our unit are now pair or group trained.

Group 4: n = 8. Some of the monkeys from Groups 1 and 2 (see Table 1) went on to be group- or pair-trained to acclimate to the primate chairs after being trained to enter t-boxes for their experiments. Group 4 provided us with the opportunity to trial whether it was possible to train primate chair naïve animals to acclimate to the chair using the training methods we had refined for acclimation to the t-boxes (see Table 1). Our measure of success in training for this group was for the monkeys to voluntarily present their head through the aperture on consecutive sessions and to hold it there for periods of at least 15 s in preparation for neck yoking while in the horizontal (sphinx) position inside the chair. The horizontal sphinx position is used for awake neuroimaging of monkeys in our primate unit. There was a time constraint of 10 days (set by the regulators) to complete this primate chair acclimation training.

Group 5: n = 7. Monkeys were group- or pair-trained to enter t-boxes as per Group 3, then immediately moved on to acclimation in a primate chair (see Table 1). Six of these monkeys also experienced neck-restraint (plate) training, as it was a requirement of the experiments to which they were assigned (see Table 1, Table 2). The other monkey in this group participated in the group-trained t-box acclimation only as this monkey was involved in a neuroimaging and neuronal tracing study under general anaesthesia that did not require neck-plate training (see Table 1).

**Table 2:** Overview of subjects showing details for each individual monkey involved in these studies including their training group for entering a transport device, their training group for neck plating, the number of weeks in unit before start of training, their age at start of training and the time taken in days to train them to acclimate to their transport device. Training group 1: individual training to enter transport device using a combination of PRT and NRT; Group 2: individual training to enter transport device using PRT only; Group 3: pair- or group-training to enter transport device using mainly PRT; Group 4: pair or group-training for neck plating using PRT and NRT; Group 5: pair- or group-training for transport device using PRT and NRT; Group 6: pair-training to enter primate chair using PRT and NRT.

Monkey	Training Group - transport device	Training Group - neck plating	Time In Unit (wks)	Age at Start of Training (wks)	Training Time (days)
M1	1		13	139	12
M2	1		13	138	12
M3	1		13	148	12
M4	1		13	149	12
M5	1		13	142	12
M6	1		13	145	12
M7	1		94	212	12
M8	Excluded from 1*		94	216	–
M9	1		69	170	20
M10	1		69	169	20
M11	1		69	162	20
M12	1		69	174	20
M13	1	4	69	197	20
M14	1		69	159	20
M15	1	4	69	179	20
M16	1		69	159	20
M17	1	4	69	180	20
M18	1	4	69	182	20
M19	1	4	69	160	20
M20	1	4	69	173	20
M21	1		69	157	20
M22	1		69	171	14
M23	Excluded from 2*	4	9	152	–
M24	Excluded from 2*	4	9	133	–
M25	2		9	153	21
M26	2		9	143	21
M27	2		9	151	21
M28	Excluded from 2*		9	149	–
M29	3		69	166	30
M30	3		39	175	11
M31	3		39	169	11
M32	3		39	156	11
M33	3		39	150	13
M34	3		1	308	11
M35	3		1	305	11
M36	3		1	308	11
M37	3		1	300	11
M38	5	5	1	259	3
M39	5	5	1	250	3
M40	5	5	1	240	3
M41	5	5	1	238	3
M42	5	5	2	266	3
M43	5	5	2	223	3
M44	6	6	2	208	3
M45	5	–	2	278	3
M46	6	6	2	220	3

* Indicates the monkeys that were excluded from their respective groups. Please see notes in the text for explanation.

Group 6: n = 2. These two monkeys, randomly chosen (by the animal technicians pulling their names out of a hat from the group of five animals on their day of arrival in the unit), were pair-trained to enter primate chairs from the beginning of their training, using the same methods and timeframes for acclimation to a t-box; these two monkeys had no prior experience of entering a t-box (see Table 1, Table 2). These two monkeys also experienced neck-restraint training as it was a requirement of the experiments they were assigned to.

### 2.2 Food control protocol for training

#### 2.2.1 Food protocol during training

Monkeys were trained to acclimate to their transport device with food and/ or fluid reward using operant and Pavlovian conditioning techniques. We also used a food control protocol. Within the home enclosure, monkeys had *ad lib* access to water. In addition, the monkeys received *controlled* access to their main, high calorific daily portion of food (wet mash and fruit), that is, they received this at the end of their daily training session. This portion of weighed food contained mash powder mixed to a dough with water and included raisins, dried banana, cereal pieces, and a tablespoon of Yumega Itchy Dog for coat enrichment. Each monkey received between 120–220 g per day of this food as a mash ball with the amount given depending on the animal’s weight (heavier monkeys received more mash). They also received at least a ½ portion of banana, and additional fruits depending on the daily selection available; including apple, orange, grape, melon, pineapple, kiwifruit, plus at least 6 dates and 6 peanuts at the end of their training session. During training to enter the t-box or chair, this food was handed to the monkey at the end of its’ training session, while it remained in the transport device. They had to eat this mash and fruit in their primate chair or transport box before returning to their home enclosure, so they were given additional time (between 10–20 min) in the transport device to finish the food or store it in their cheek pouches. Furthermore, if the monkey dropped its food while in the transport device that then became out of its reach then the trainer handed the monkey this food again, as it is a requirement of the Home Office Project Licence. The monkeys learned to associate coming out of their enclosure into the transport devices with receiving their daily ration of high calorific mash and fruit at the end of each training session. Upon returning to their home enclosure after their training session, the trainer offered the monkey up to 4 peanuts or dates. As a condition of the Home Office Project Licence that authorised this monkey research work, we weighed each monkey at least once per fortnight to record and adjust their food portions accordingly.

#### 2.2.2 Food protocol in home enclosure

At other times during the day, within the home enclosure, all monkeys received forage mix, which was distributed amongst the wood shavings substrate on their floor, or by means of different types of enrichment devices (Honess and Marin, 2006). This dried forage mix (see Appendix A), comprised of a mixture of cereals and seeds, and was given up to twice a day when monkeys were not participating in daily testing sessions (e.g. on their days off training). When monkeys were participating in testing sessions then, typically, we limited the distribution of forage to once per day (i.e. if a group of monkeys were working in the morning, the forage was provided in the afternoon otherwise it was provided in the morning if they were working in the afternoon). On Friday evenings, all monkeys received a forage bag or box containing popcorn, hay, and different cereals, as well as forage mix. Other low calorific foods (e.g. sticks of celery, button mushrooms, florets of broccoli and cauliflower, snow peas, or frozen ice cups with seeds inside the frozen water/ diluted juice) were also provided in the home enclosures at times throughout the day when the monkeys were not involved in training.

When monkeys were not being trained, the animal care technicians were requested to provide the animals with their entire food intake for the day. Typically, on the monkey’s days off training, the animal technicians would provide their wet mash in the morning and their portions of fruit in the afternoon. Sometimes it was necessary to separate the monkeys into smaller groups or individually while they were eating their main meal when in their home enclosures to ensure that all monkeys ate their daily ration. Also if for some reason it was not possible for the researcher or animal trainer to test their monkeys during the day as expected, then an animal technician or a colleague was asked to give the monkeys’ their food, rather than getting the regular primate trainer to feed them in their home enclosure. These additional methods for feeding the monkeys when they were not working helped to establish the association that when the monkeys saw their regular trainer, they knew that it was a day for coming out of their home enclosure for a training session, and that they received their main meal while inside their transport device after this session was completed.

#### 2.2.3 Use of peanuts as rewards

In addition, we controlled the distribution of dates and peanuts and other palatable treats to our monkeys. Other labs have also now reported similar practices (Martin et al., 2018). We believed these food items are a key motivator for the monkeys during their training sessions, so we used them as rewards during training only. We specifically requested that our animal technicians or veterinarian staff did not give the monkeys these rewards in their home enclosures. Instead, they used raisins, which are also highly palatable food rewards - but different to the ones we used for training - when performing their daily health and welfare checks, or when moving the monkeys around inside their home enclosures for husbandry purposes.

### 2.3 Training methods

Our training methods used PRT only or combined PRT and NRT. PRT for these experiments involved the presentation of treats (fruits, raisins, peanuts, or a diluted juice liquid reward) for desired behaviors when produced at the command signal (stimulus). We used ‘over’, ‘down’, and ‘in’ to move the monkey around inside their home enclosure and enter the transport device. The command signal ‘head’ or ‘up’ was used to encourage the monkey to present their head through the primate chair aperture.

We also used a neutral stimulus, like a hand clicker, voice click, whistle or a phrase (e.g. ‘good boy’) as a ‘bridge’ (also referred to as a secondary reinforcer) that was kept consistent for the monkeys, to signal to each monkey that it had produced a desired behavior and that a food reward would be coming in due course. This ‘bridge’ method incorporates the principles of classical conditioning (Mellen and Ellis, 1996). The sound (e.g. voice click or phrase) was especially helpful to use during the manipulation of the primate chairs or t-boxes when our hands were unavailable to operate a clicker or present treats. The ‘bridge’ is also obviously used only after a period of time in training when the monkey had been conditioned to understand that this previously neutral stimulus now signalled that they had performed a desired behavior and that a palatable reward would soon be presented (Mellen and Ellis, 1996).

In addition to these PRT techniques, we also sometimes needed to use NRT techniques. It is critical to know that PRT must be used first and most often in order to train the monkeys. We have found that the use of PRT helps to establish a positive relationship with a monkey. However, sometimes it was necessary to use NRT techniques as well. NRT techniques must be used quickly and effectively to encourage the monkey to present a desired behavior with the use of the command signal so that the monkey can avoid the noxious reinforcer (e.g., the net, or the winding handle of the squeeze-back being used to reduce the space – see below), which then ‘disappears’ when the target behaviour is performed. It is very important to follow the NRT technique with a positive reinforcer (e.g. the secondary ‘bridge’ reinforcer, or a reward) to further establish the relationship among the stimulus (i.e. the command signal), the desired behaviour, and the reinforcement (initially for NRT this will be the disappearance of the net, eventually followed by the receipt of some dates or peanuts). The NRT reinforcer must be removed quickly, once the desired response is produced, otherwise the monkey may associate the presentation of a NRT reinforcer with its ability to receive rewards and then the NRT reinforcers will become ineffective. In addition, after the use of a NRT reinforcer with one monkey, it must be removed from sight of the other monkeys. We kept our nets in a cupboard along the main colony corridor outside of the home enclosures and the detachable winding handle outside of the home enclosure rooms.

For us, NRT reinforcers also involved the use of the retractable rear walls (squeeze-back) permanently located at the backs of some of the cages in the monkeys’ enclosures. These cages (‘out-pens’) were where the monkeys exited from their home enclosures. These retractable walls could be moved forward to encourage the monkey to come towards the front of the out-pen of their home enclosure, via a winding mechanism. It was important the monkey was offered a treat when it came to the front and that the ‘bridge’ stimulus was used while it was coming forward so that the monkey understood that this was the desired behavior that would lead to reward. We would like to note that the retractable walls were also used for husbandry and veterinary purposes by the care staff, and monkeys had experience of this NRT reinforcer on a regular basis. In addition, the retractable walls in the home caging were also installed and used in the UK breeding centre, from where we sourced our monkeys. We also noted that some of the monkeys became conditioned to understanding that the presentation of the detachable winding handle could result in the reduction of the space inside their primate closure, and in most cases, the presentation of the winding handle alone (i.e. without reducing the space) was also sufficient as an effective NRT reinforcer. We also sometimes needed to present a handling net to a monkey inside its’ home cage to encourage the monkey to move across into the out-pen of its’ home enclosure. It must be noted though that some NRT reinforcers will cause the monkeys to freeze instead of presenting a different behavior, or they will not be bothered. If necessary, we presented a NRT reinforcer to a monkey to determine if it would elicit a different response, we then removed it quickly and used the ‘bridge’ stimulus and offered treats to encourage the monkey to move to its’ out-pen or come into the transport device. When inside the primate chairs, space-reducing rear and side panels were also used during neck-restraint training for some of our monkeys (see Table 3 and Section 2.3.1 below). These may be considered as a NRT reinforcer. Similarly, once the desired response was produced (i.e. presenting their head through the aperture for neck-plating), the NRT reinforcer was removed immediately.

**Table 3:** Use of additional methods required per training day to reposition the monkey inside the primate chair so that it could voluntarily present its head for neck-plating. The additional methods used are decoded by means of 0 (not used) and 1 (used). First value, in columns Session 1 to Session 10 indicates use of rear reducer, second value, the use of side reducers, and third value, moving to horizontal position. For the first two sessions (Session 1 and Session 2) no additional methods were used.

Name	Group	Session 1	Session 2	Session 3	Session 4	Session 5	Session 6	Session 7	Session 8	Session 9	Session 10
**M37**	5	0 0 0	0 0 0	1 1 1	1 1 0	1 0 0	1 0 0	1 0 0	1 0 0	0 0 0	0 0 0
**M38**	5	0 0 0	0 0 0	1 1 1	1 1 1	1 1 0	1 0 0	0 0 0	0 0 0	0 0 0	0 0 0
**M39**	5	0 0 0	0 0 0	1 1 0	1 0 0	1 0 0	0 0 0	0 0 0	0 0 0	0 0 0	0 0 0
**M40**	5	0 0 0	0 0 0	1 1 1	0 0 0	1 0 0	0 0 0	1 0 0	0 0 0	0 0 0	0 0 0
**M41**	5	0 0 0	0 0 0	1 1 1	1 1 1	1 1 1	1 1 1	1 0 0	1 0 0	0 0 0	0 0 0
**M42**	5	0 0 0	0 0 0	0 0 0	0 0 0	0 0 0	0 0 0	0 0 0	0 0 0	0 0 0	0 0 0
**M43**	6	0 0 0	0 0 0	0 0 0	1 1 1	0 0 0	1 1 1	1 0 0	0 0 0	0 0 0	0 0 0
**M45**	6	0 0 0	0 0 0	1 1 1	1 1 1	1 0 0	1 1 1	1 0 0	1 0 0	1 0 0	0 0 0

It is important to emphasize that clear definitions and training goals must be decided upon by the trainer to determine what is the specific desired behaviour so that they know when to reward the monkey consistently. The trainer must always immediately offer rewards or use the ‘bridge’ stimulus (when the monkey understands what this means) when desired behaviors are displayed so that the monkey can associate what it has just done with a rewarding outcome. It is also the case that a reward or the ‘bridge’ stimulus must be provided if the desired behavior was elicited via the use of an NRT technique. Further, even when a monkey is well acclimated to its transport device and is neck-plate trained, there may be periods of regression that require the use of NRT techniques over the course of its study duration.

#### 2.3.1 Additional use of methods for neck-restraint training in the primate chair

**Fig. 1:**
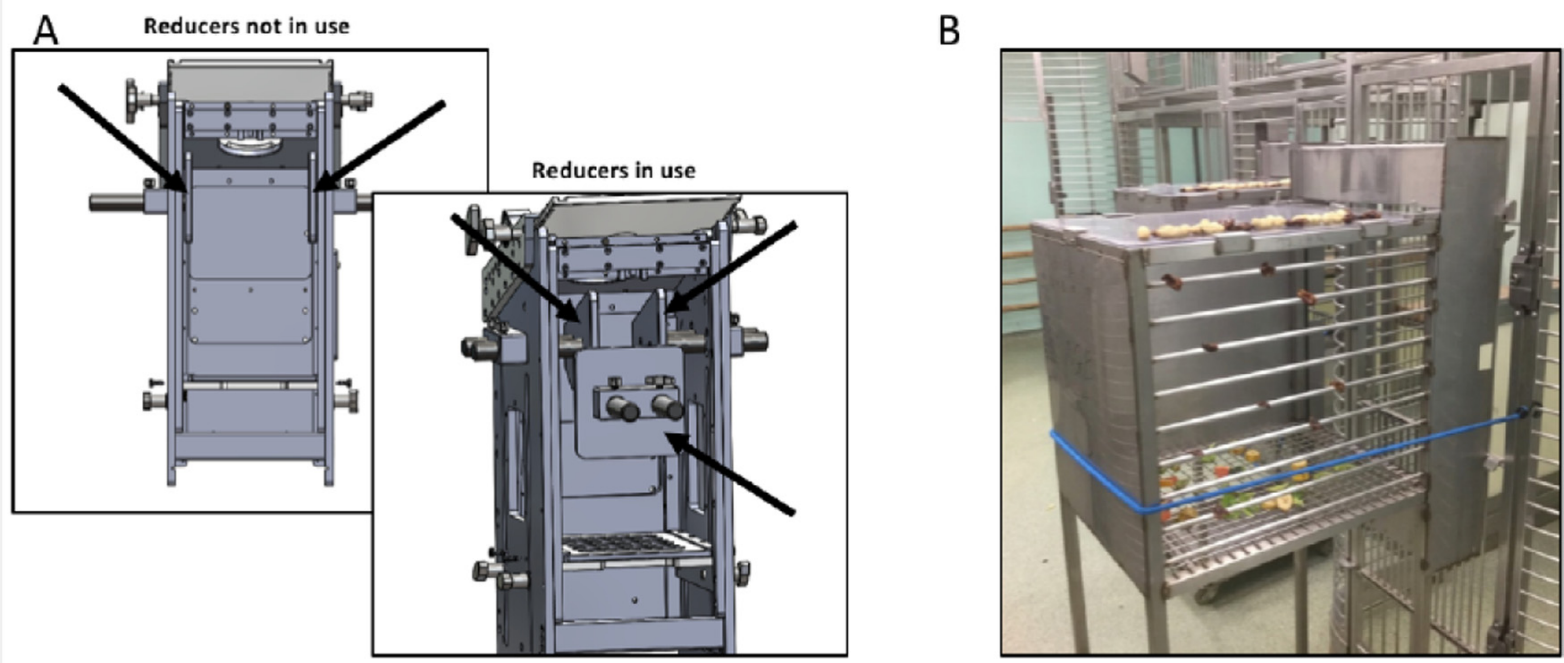
Methods. A. Back of our primate chair, designed and manufactured by Hybex Innovations, distributed by Rogue Research Inc. The primate chair shows the modifications that were designed in conjunction with our lab at Oxford from 2013 to include side and rear reducers (indicated by arrows). B. Custom built transport box attached to home cage.

In addition, after the monkey was in the primate chair, the use of side and rear space reducers in the chair (an additional NRT reinforcer; see Fig. 1A) or moving the primate chair from the vertical to the horizontal position were also effective for encouraging the monkey to move into a different position that allowed it to see the highly palatable reward. The operation of the side and rear reducers inside the primate chair has been documented in this video [https://view.vzaar.com/19365872/video]. The use of these space reducers encouraged the desired behavior (voluntarily presenting its head through the chair aperture) during training so that the animal was able to succeed in receiving its highly palatable rewards during the training session. These additional methods were especially helpful in the initial stages of neck-plate training or could be used during periods of regression during the course of the experiment.

Finally, we believe that contextual cues were also key to help facilitate a positive experience for the monkey, e.g. the colour of the lab coat used by the trainer. In Oxford, the colour green signified a normal training day, while purple clothing worn by the staff indicated a general anaesthesia procedure was occurring and that the retractable walls inside the primate caging would be used in order to restraint the monkey to receive an intramuscular injection for sedation. In addition, at the beginning of a training session, we entered the home enclosure room while wheeling the primate transport device (t-box or primate chair) to help the monkey associate that seeing their transport device meant that it was coming out for a training session.

### 2.4 Monkeys’ arrival in facility

All monkeys that participated in these experiments were bred in the UK Centre for Macaques breeding facility. Upon arrival, the monkeys were housed together in their group in a quarantine room. The home enclosures were baited with forage before the monkeys’ arrival, and they were presented with their food biscuits (standard diet at the breeding facility) along with a piece of fruit by the animal technicians. During this first week of arrival, the monkeys experienced their initial acclimation to their primate trainer (as detailed below) followed in Week 2 by training to acclimate to their transport device as per the details described below.

#### 2.4.1 Week 1 – during the week of the monkeys’ arrival in the unit

The trainers got to know their monkeys by interacting and talking with them and giving them treats for approaching the front of their home enclosures and for moving across into the different pens (i.e. using PRT training techniques). We also introduced the use of a clicker, or whistle as a ‘bridge’ stimulus to the voice command ‘over’. Importantly, we paired the ‘bridge’ (neutral stimulus) with the presentation of a food reward (primary reinforcers; an example of classical conditioning), thus the use of the clicker, or whistle, or other ‘bridge’ stimulus started to become a conditioned stimulus (CS). Once the monkeys had established the association, we used the ‘bridge’ stimulus, as a secondary reinforcer, to ‘reward’ desired behaviors, in addition to, or sometimes instead of, offering them the primary (food) reinforcers.

##### 2.4.1.1 Week 2 – acclimation to the transport device (t-box)

Sessions 1 and 2: Several t-boxes were attached to the front of the home enclosures with bungee cords to hold them in place (Fig. 1B). Note that at this point, all monkeys were grouped in the out-pens. It was important to use several t-boxes so that the more dominant monkeys would not block the access of the less dominant monkeys. Fruit and treats were positioned on the insides and on the tops of the t-boxes, and the monkeys were able to enter the devices and eat these food rewards (Fig. 1B). The monkeys were also presented with their daily mash food while they were coming into or touching the transport devices.

Sessions 3 and 4: The monkeys were separated into smaller groups within the out-pens, and a t-box was attached to the front of the enclosure (as before). Highly desirable rewards, like peanuts and raisins, were positioned on top of the t-box. As soon as one monkey entered the t-box, it collected the rewards from the top of the t-box, which allowed time to close the door. The monkey was then wheeled away from the front of the home cage enclosure to allow the next t-box to be setup in its place. The monkeys received ½ banana as soon as they entered the device, followed by another ½ portion of fruit, plus their daily mash. The trainer also handed them several peanuts and/or dates and continued to talk to them in a reassuring tone. During the training in groups or pairs, all monkeys were removed from the ‘out-pens’ one after the other, and sat together in their separate transport device while they ate their mash and fruit.

Session 5: Once the monkeys entered the t-boxes, the t-box doors were closed and they were wheeled out of the home enclosure room to an adjacent area in the corridor just outside their home room. While in the corridor, the trainer was continually interacting with them and they received their mash, their fruit and additional highly palatable rewards. In order to enter the t-box, highly palatable food rewards were given and these were typically individualized for each monkey around this time as by this stage each monkey had shown a preference for peanuts, dates or another highly desirable food (e.g. banana). It is important to monitor the individual monkey’s preferences so that the rewards offered for desirable behaviors are tailored to the individual monkeys appropriately.

##### 2.4.1.2 Week 2 – acclimation to the transport device (primate chair)

Sessions 1 and 2: Several primate chairs were attached to the front of the home enclosures with bungee cords to hold them in place. All monkeys involved in the chair acclimation were grouped together in the out-pens. It was important to use several primate chairs so that the more dominant monkeys would not block the access of the less dominant monkeys. Fruit and treats were positioned on the floors of the chairs and also at the top of the chair aperture, and the monkeys were able to enter the devices and eat these food rewards. The monkeys were also presented with their daily mash food while they were coming into or touching the primate chair.

Sessions 3 and 4: The monkeys were separated into smaller groups within the out-pens, and a chair was attached to the front of the enclosure (as before). Highly desirable rewards, like peanuts and raisins, were positioned at the aperture on the top of the chair. As soon as one monkey entered the chair, the door was closed. The monkey was then wheeled away from the front of the home cage enclosure to allow the next chair to be setup in its place. The monkeys received ½ banana as soon as they entered the chair, followed by another ½ portion of fruit, plus their daily mash. The trainer also handed them several peanuts and/or dates and continued to talk to them in a reassuring tone. During the training in groups or pairs, all monkeys were removed from the ‘out-pens’ one after the other, and sat together in their separate chair while they ate their mash and fruit.

Session 5: This was conducted in the same manner as the t-box training (see Section 2.4.2.1).

### 2.5 Week 3 – acclimation to the transport device (t-box or primate chair)

Session 1: Involved a repetition of Session 5 from Week 2.

Session 2: The monkeys entered the t-boxes or primate chairs. They were wheeled along the unit corridor to a suitable separate room. While in this room, in either their groups or pair, they received their mash and fruit and additional highly palatable rewards.

Sessions 3 - 5: The monkeys were wheeled in their t-boxes or primate chairs to the experimental setups. At this stage the monkeys were separated into individual testing cubicles. A stainless steel ‘lunch box’ located in front of the computer touchscreen (on which eventually the tasks were presented) was filled with their mash, fruit and peanuts and dates, and was opened upon the monkeys’ arrival.

### 2.6 Additional methodology for acclimation to the primate chair

We used two different approaches to explore optimal methods to acclimate our monkeys to our primate chairs; these chairs were used for awake neuroimaging, which requires the horizontal sphinx position, or neurophysiological recordings, which requires the vertical sitting position (Fig. 1A). For the two approaches, the monkeys used in these studies either were previously trained to enter the t-box, and were then trained to enter the primate chair voluntarily according to the procedures detailed above (in Section 2.4.2.1 and Section 2.4.2.1; Groups 4 and 5), or were monkeys that had not previously received t-box training and thus were only trained to acclimate to the primate chairs (Group 6) again using the same training protocol as detailed above but only involving the primate chair methodology described in Section 2.4.2.2. For all primate chair acclimation, we only worked with the monkeys in groups or in pairs.

### 2.7 Neck restraint training

Neck restraint or neck plate training involved the monkey having its head held outside of the primate chair. The neck-plating device did not restrict the monkey’s ability to move its head or its neck (i.e. the monkey could still turn around completely inside the primate chair while it was neck plated). Transparent plastic plates attached to the sides of the aperture were secured to make the aperture in the chair too small for the monkey to retract its head back down inside the chair. For our neuroscience experiments that require the primate chair, the monkey’s head needs to remain outside of the chair so that appropriate recording devices or neuroimaging coils can be fitted to the monkey’s head, and it can see the touchscreen stimuli and participate in the experiments.

The training schedule for voluntary neck plating typically started on the first session of the training week to allow for at least five consecutive sessions of training.

Session 1: Monkeys had voluntarily entered the primate chair, which was positioned vertically. The neck-plate was then opened allowing the monkey to voluntarily present its head up through the aperture and out of the top of the chair. The movable base (floor) plate inside the chair was adjusted to make sure the monkey could do this comfortably. During this training session, we positioned an open food container filled with highly palatable food rewards so that it was visible to the monkey. Inside the container was their favorite fruit, cut up into long pieces, peanuts and dates, and diluted blackcurrant cordial, presented to the monkey’s mouth via a plastic syringe; these rewards were offered to the animal to encourage the desired behavior (i.e. head presentation). Importantly, when offering the treats we would typically target the tongue of the monkey, as this would encourage them to present their head forward and up. In this first session, the monkeys were rewarded for presenting their heads up through the aperture, but the neck plate was not closed. Any remaining treats were offered to the animal at the end of the training (while the animal was still sitting inside the chair). We also found that it was preoccupying for the monkeys for the trainer to shell their peanuts in front of them while they were presenting their head out of the top of the chair and we encouraged them to lick the peanuts directly out of the opened shell.

Session 2: The same routine was followed for the second session, except that the neck plate was closed as soon as the monkey presented its head out of the top of the aperture. Typically, the monkeys would respond to having the neck plate closed in several different ways, by either, doing nothing differently, or they began to turn around in their primate chair, or they made cooing noises, or sometimes they would start grinding their teeth, or yawning. After the neck plate had been closed, the trainer sat quietly beside the monkey, calmly talked to the monkey, and used their voice and the ‘bridge’ conditioned stimulus as a secondary reinforcer for desired behaviors (e.g. when the monkey was displaying periods of calmness). We allowed the monkey to acclimate to this new situation and offered them food and fluid rewards (e.g. diluted blackcurrant cordial) as detailed above for approximately 5 min or until the monkey’s cheek pouches were full. Any remaining food from the open plastic container that the monkey could see was presented to the monkey after the neck plate training session was completed (i.e. after the monkey had the neck plate released and it had withdrawn its head back inside the primate chair). Critically this remaining food plus their mash was given to the monkey and time was allowed for it to eat it and store the rest in its cheek pouches while it was still seated inside the primate chair.

Session 3: Repeat as per above. Typically, in our experience, some (but not all) of the monkeys were not inclined to participate in the neck plate training during Session 3. To encourage the monkey to present its head again voluntarily and to be neck plated, we adjusted the monkey primate chairs to include side and rear space reducers (Fig. 1A and as displayed in this video link [https://view.vzaar.com/19365872/video]). If the monkey was not keen to present its head voluntarily for neck plating, (i.e. it would sit off to one corner of the chair with its head lowered near its abdomen) then we would use the side and/or rear reducers in the chair to reposition the monkey with its head above the aperture so it could see the palatable rewards and poke its head out to receive them. The ‘bridge’ stimulus was used, and food rewards offered when desired behaviors were displayed. The side and/or rear reducers were released as soon as the monkey was neck plated and further food rewards were offered immediately.

Finally, it was also sometimes useful to re-position the chair in the horizontal (sphinx) position in order to encourage the monkey to present its head out of the aperture, now located on the side of the chair when in the horizontal position. Similarly, the ‘bridge’ stimulus and food rewards were offered when desired behaviors were displayed. As soon as the animal was neck plated, the primate chair was carefully repositioned vertically and many highly palatable food and fluid reward treats were immediately offered to the monkey.

Sessions 4 - 6: The training sessions were repeated as per above. As the monkeys learned what was required in their training sessions and voluntarily presented their head for food and fluid rewards using PRT and the ‘bridge’ stimulus, the use of the side and or rear reducers, and or repositioning in the horizontal position were removed.

Session 7: The monkeys were moved to the experimental setup to begin their neuroscience research studies.

### 2.8 Statistical analysis

Non-parametric statistical testing was used (SPSS version 24), as the data were categorical, including Wilcoxon rank sum, Kruskal Wallis, and Friedman’s tests and Spearman’s *rho* rank order correlations, with the significance level set to *p* <  0.05 (two-tailed). All post hoc pairwise comparisons used a Bonferroni correction. In addition, we rounded up to the next whole day for analyses that involved means and standard deviations of days to complete acclimation to the transport device.

## 3 Results

**Fig. 2:**
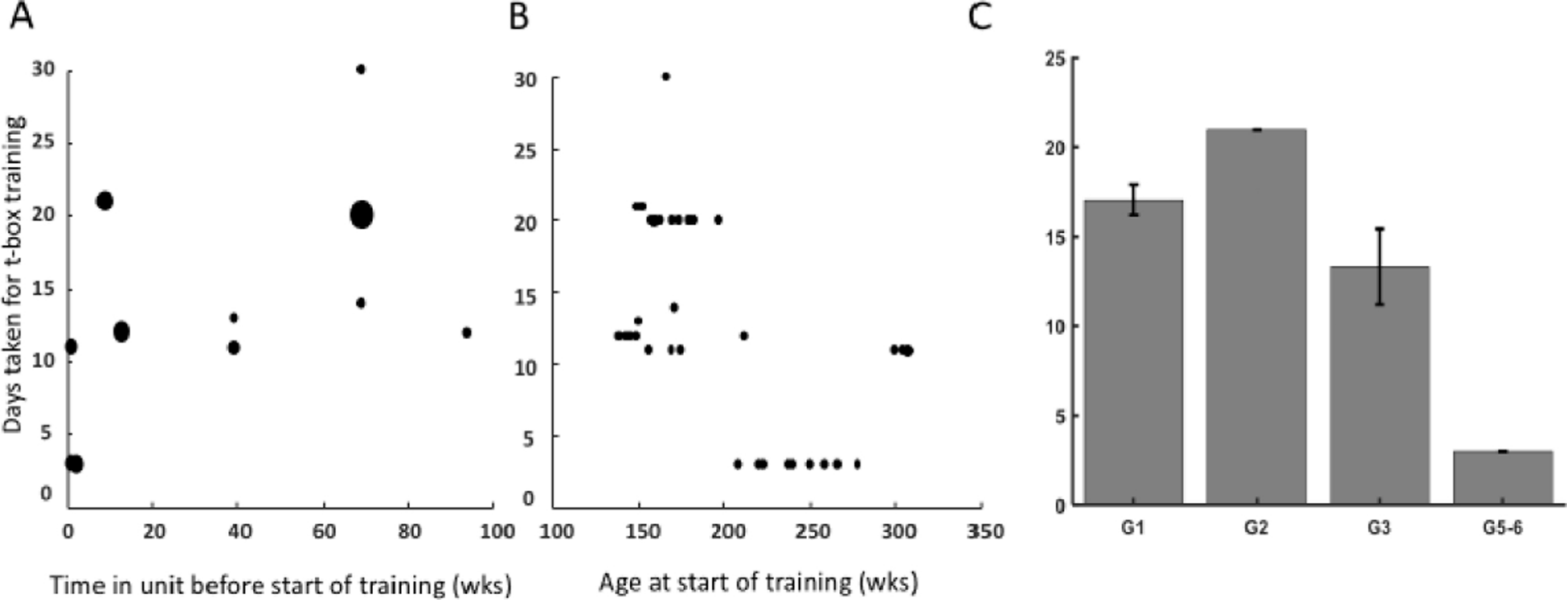
Days taken for t-box or chair training. A. As a function of time in unit before start of training. B. As a function of age at start of training. Dot thickness represents number of measurements. C. As a function of training group.

Over the period of the 9 years of data collection, 46 monkeys arrived in the Oxford primate unit and either immediately began to participate in neuroscience experiments under the licence of Dr AS Mitchell, or were initially involved in immunology studies prior to transferring to the licence of Dr AS Mitchell in order to participate in our neuroscience experiments (see Table 1, Table 2). Thus, given that different monkeys started their training at different times after arriving in the unit, it was possible to analyse the differences in the number of weeks since an individual monkey had arrived in the primate unit and the time taken to acclimate it to its transport device. The positive Spearman’s rank-order correlation between number of weeks in the unit and time taken to acclimate to the transport box, [rho = 0.71, n = 42 (Group 1–6), *p* <  0.001] (Fig. 2A), indicated that the sooner training began after arriving in the unit the less time it took to acclimate an individual monkey to the transport box (mean time spent in unit before training starts: 33.4 weeks (range 1 week to 94 weeks), SD: 31.3 weeks; mean acclimation time: 14 days, SD: 8 days). We started working with the monkeys in Groups 5 and 6 (group- or pair-trained using PRT and NRT) immediately upon arrival. For the two monkeys in Group 6 that were trained to acclimate to the primate chair from the beginning of their training, it took them 3 days to acclimate, which is smaller than the range of 95% CI ([11.89–16.15]), confirming that it was more optimal to begin training as soon as possible upon arrival. These two monkeys in Group 6 had arrived in the unit with some of the monkeys in Group 5 and followed the same training protocol, except that the monkeys from Group 6 were trained to enter and acclimate to a primate chair instead of a t-box. The monkeys in Group 5 were also quicker to acclimate to the t-boxes on average (average number of days: 3; SEM: 0) compared to the monkeys in Group 1 (individually trained using PRT and NRT), Group 2 (individually trained using PRT only), and Group 3 (pair- or group-trained using PRT and NRT), who had started their transport device acclimation up to many weeks (range 1–94 weeks) after arrival in the unit (Table 2).

In addition, it was possible to establish if there was a correlation between how old the monkeys were at the beginning of their training and the time taken to acclimate to the transport device (see Table 2). This analysis revealed a negative significant correlation co-efficient, [rho = -0.557, n = 42 (Group 1–6), *p* <  0.001] (Fig. 2B), indicating that the older the monkeys were the less time it took them to acclimate to their transport device, although our oldest monkeys were still only between 5 and 6 years of age when they began their transport device acclimation training.

### 3.1 Initial primate transport device acclimation

As described in the training methods section above, the monkeys were divided into four different groups (Group 1, 2, 3, and 5/6) based on various conditions used to explore optimal methods for acclimating the monkeys to their transport device. Group 4 were excluded from this analyses (see Methods for details). Group 1 received individual training, combining PRT and NRT; Group 2 received individual training with PRT only, i.e. no NRT was used; Group 3 were group trained, mainly using PRT but some NRT was required for 1 monkey; and Group 5/6, the monkeys were group trained, combining both PRT and NRT, but mainly and always PRT was used first. The three monkeys from Group 2 that did not acclimate within 21 days using PRT only were not included in this analysis, nor was the one monkey from Group 1 that had taken many weeks to approach the trainer and touch or sit in the t-box (as per the details provided in the Subject Methods section), leaving N = 42. The non-parametric Kruskal-Wallis test revealed a significant difference in the total number of days taken to acclimate to the transport device as a function of Group [H = 30.90, df = 3, *p* < 0.001] (Fig. 2C). Further post hoc pair-wise comparisons amongst the groups revealed a significant difference (t-stat = 22.67, *p* <  0.001) between Group 1 (mean: 17 days, SD: 4 days) and Group 5/6 (mean: 3, SD: 0), with Group 1 taking more days to acclimate to the t-box; between Group 2 (mean: 21 days, SD: 0) and Group 5/6 (t-stat = 35.00, *p* <  0.001), with Group 2 taking more days to acclimate to the t-box; and between Group 2 and Group 3 (mean: 14 days, SD: 7 days) (t-stat = 22.56, *p* <  0.028), with Group 2 taking more days to acclimate to the t-box.

### 3.2 Primate chair acclimation

A group of 8 monkeys (Group 4) were acclimated to enter a primate chair at the end of their neuroscience experiments; they had all been previously trained to enter t-boxes and had originally been in either Group 1 or 2 (see Table 1, Table 2). The monkeys were trained for a total of ten sessions to voluntarily enter a primate chair and raise their head through the aperture for at least 15 s per training session using only PRT, and we recorded the time that the required behavior was performed.

#### 3.2.1 Primate chair training – (Group 4: Sessions 1 and 2)

**Fig. 3:**
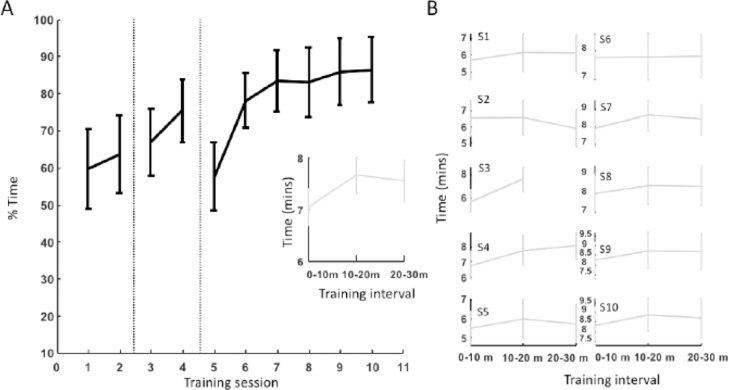
Group 4: Chair exploration and head presentation. A. Average percent of time spent on the desired response per training session. Black bars indicate SEM over subjects. Session 1 and 2: chair exploration. Session 3 and 4: Head presentation in vertical position. Session 5–10: Head presentation in horizontal position. Inset: Average time (in minutes) over all sessions spent on the desired response per 10 min interval. Vertical bars indicate SEM over sessions. B. Average time (in minutes) spent on the requested behavior per 10 min interval, per training session (Session 1 – Session 10). Vertical bars indicate SEM over subjects.

During the first two training sessions (1 and 2) for Group 4 monkeys, they were rewarded using PRT when they approached or entered the chair; and all monkeys succeeded at entering the chair within two sessions. Note that the monkeys were already extensively trained to enter a t-box. We recorded the amount of time the monkeys spent touching, or sitting in the chair in a 30 min period (divided into three 10 min intervals) (Fig. 3). On average, monkeys spent 17.9 min (60.0%, SEM: 3.2) interacting with the chair in Session 1, and 19.0 min in Session 2 (63.4%, SEM: 3.1; Wilcoxon signed rank test between Session 1 and Session 2: 8, *p* =  0.20). Importantly, longer training sessions did not affect the required behavior, as the time spent on the required behavior (i.e. approaching or entering the chair) did not change over the 30 min testing session (Fig. 3B, Friedman test on the effect of interval: Session 1: x2(2) = 3.19, *p* =  0.2; Session 2: x2(2) = 2, *p* =  0.38. Linear regression: Session 1: slope = 0.21, *p* =  0.4; Session 2: slope = -0.3, *p* =  0.34). This result therefore supports keeping the training sessions relatively short, i.e. no longer than 30 min.

#### 3.2.2 Head presentation (Group 4: Session 3–10)

In Session 3, during the first training day that the monkeys had the door closed on the primate chair, they voluntarily presented their heads. We recorded the time the monkeys presented their heads in a 20 min interval (divided in 2 x 10 min intervals). Note that the training session on Session 3 was deliberately shorter compared to the other training days due to the possibly stressful experience of being inside the chair and away from the home cage for the first time. On average, the monkeys presented their heads for 13.3 min (SEM: 1.8) of the recorded 20 min interval (66.7%, Fig. 3A), with a small increase in the second 10 min interval (Wilcoxon signed rank: 2, *p* =  0.02, Fig. 3B).

The monkeys significantly increased the amount of time spent voluntarily presenting their heads between Session 3 and Session 10 (Fig. 3A, Friedman test on the effect of session, x2(7) = 20.37, *p* =  0.005), with 7/8 monkeys presenting their heads more than 90% of the time on Session 10. Data on Session 3 and 4 were collected while the monkeys were presenting their heads with the chair positioned vertically, while on Session 5–10 the chair was moved to the horizontal sphinx position. Although the time spent presenting their heads decreased slightly (but non-significantly) for Session 5 (17.3 min, SEM: 2.7, 57.6%) compared to Session 4 (22.6 min, SEM: 2.5, 75.4%; post-hoc Wilcoxon signed rank: 32, *p* =  0.05, Fig. 3A), the monkeys quickly recovered and displayed the same behavior on Session 6 (23.4 min, SEM: 2.2, 78.0%) compared to Session 4 (post-hoc Wilcoxon signed rank: 9, *p* =  0.25). The latter indicates that the training remained constant even in changing circumstances.

In addition, we established whether increasing the length of time of a training session affected the amount of time spent on the required behavior (i.e. head presentation; average over Session 4-10: Friedman test on the effect of interval, x2(2) = 11.14, *p* =  0.004), but only between the first and second 10 min interval (Fig. 3A, inset; post hoc Wilcoxon signed rank: Interval 1 - Interval 2: 0, *p* =  0.02; Interval 2 – Interval 3: 20, *p* =  0.38; Fig. 3B for individual training sessions). This evidence shows that keeping training sessions shorter (i.e. less than 30 min supports successful training) as the desired behaviors were presented within the first 10–20 min of the session. In addition, our training sessions were conducted on consecutive week days, so shorter training sessions ran on a daily basis are most effective.

Overall, our results indicate that a rhesus macaque can be trained to voluntarily enter the chair and present its head through the aperture (without it being closed) in less than 10 consecutive training sessions, with one training session lasting no longer than 30 min. Furthermore, increasing the duration of a training session does not necessarily elicit better performance towards the end of the session. Finally, monkeys generalized the trained behavior even across changing circumstances (e.g. vertical vs horizontal chair position).

### 3.3 Neck-plating in Groups 5 and 6

**Fig. 4:**
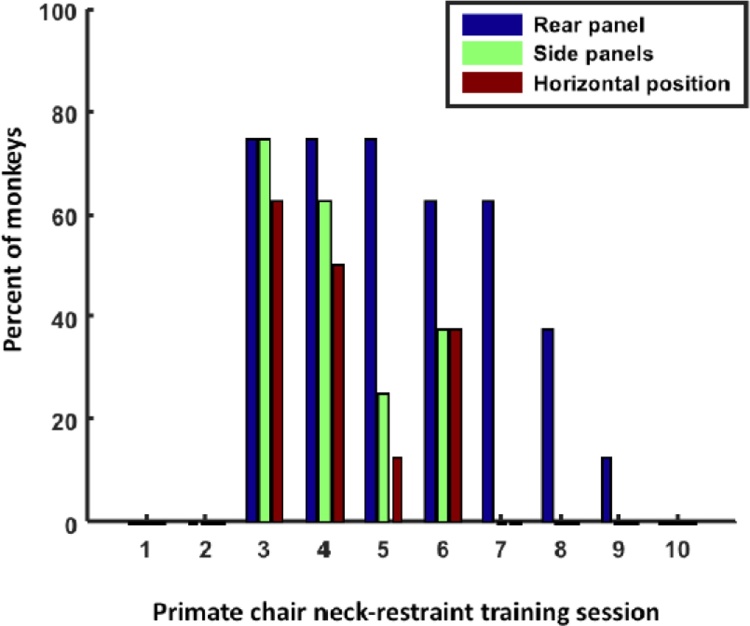
Groups 5 and 6: Neck-plating data. Plot represents the percentage of NHPs for which additional methods had to be used (rear reducer, side reducers, horizontal position) per training session.

Eight monkeys from Groups 5 and 6 were included in this study (see Table 1, Table 2 for details). All of the monkeys had initially acclimated to the primate chairs in 3 sessions of training (as detailed above). We then trained the monkeys to acclimate to neck-plating, and calculated the number of additional methods used per training session while neck-plating the animals. In Session 2 of neck-plate acclimation, all monkeys were successfully neck-plated without any space reducers used (Fig. 4). However, from Session 3, after their first experience of neck-plating in Session 2, 6 of the 8 animals would not voluntarily present their head through the aperture, thus the space reducers were used (see Table 3). Note that the space reducers may be considered to be a NRT reinforcer, and we treated them as such (i.e. we used the voice command signal of ‘up’ or ‘head’ and presented the NRT reinforcer and then removed the reinforcer as quickly as possible after the desired response had been produced and still offered rewards afterwards). As a first measure, we used the rear-reducer (this takes 20 s to implement as a separate handle is required to operate it but only a few seconds to remove), followed by the side-reducers (these take about 5 s each to implement and remove as they are already attached to the chair). Sometimes we also found it was effective to move the primate chair from the vertical to the horizontal position (this takes up to 1 min to implement). Fig. 4 shows that the majority of the monkeys needed all three measures (space reducers and horizontal positioning) in Session 3 (5/8 subjects, 62.5%) to be able to successfully neck-plate them. Importantly, 2/8 monkeys (25%) could be neck-plated using only PRT even in Session 3. Furthermore and critically, we found a rapid decline in the percentage of monkeys requiring the space reducers from Session 3 to Session 10 (linear regression on mean: intercept: 100.45, *p* <  0.001; slope: -10.57, *p* <  0.001), indicating that this method was effective to support them to succeed in their training and receive the highly palatable rewards. At Session 7, only the rear reducer had to be used (62.5% of subjects), and again a percentage drop occurred in the next two days to 12.5%. In Session 10, all monkeys voluntarily presented their heads for neck-plating without the use of any space reducers. All of the monkeys continued to present their heads voluntarily for neck-plating from Session 10 onwards with no regression or need for further use of NRT techniques.

In our training procedures, typically the monkeys were initially trained to enter a t-box for experimental or husbandry requirements. This is, however, not a typical design in other primate research centers, where the monkey will immediately be trained to enter the primate chair and will receive all their training inside the primate chair. Thus two monkeys in Group 6 were never habituated to the t-box and instead were immediately trained to enter the primate chair using the same methods for t-box acclimation. Similar to the other 6 monkeys from Group 5, who followed our usual routine of acclimation to the t-box first, these two monkeys from Group 6 were also voluntarily presenting their heads for neck-plating at Session 10 (Fig. 4). Note that linear regression did not lead to a significantly negative slope (-9.92, *p* = 0.1), most likely due to the limited number of monkeys included (slope for 6 transport-box trained monkeys: -10.78, *p* =  8.2057e-05).

**Fig. 5:**
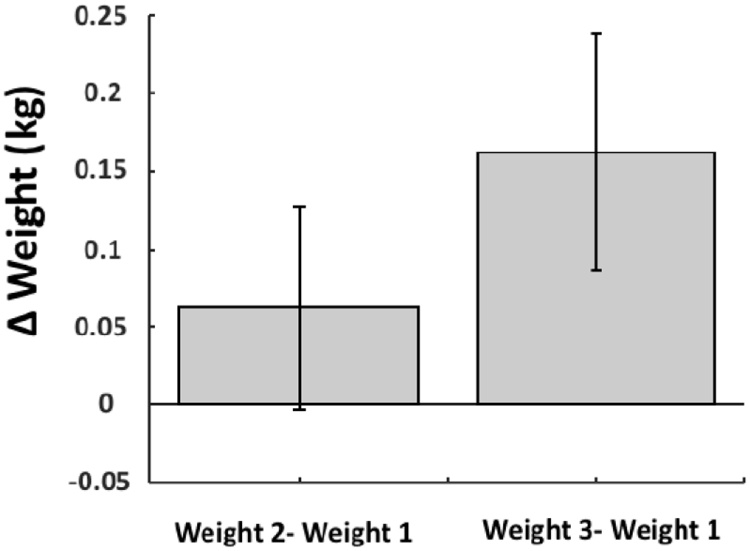
Weight. Average weight difference before training (measured between 4 and 0 days before training) and one week after training (7–8 days), and average weight difference before training and two weeks after training (14–20 days after training). Black lines indicate standard error of mean.

Importantly, the monkeys received their full high calorific daily ration of food (fruit + wet mash primate chow) and extra liquids while they were seated in the primate chair but not neck-plated. The latter did not reflect negatively on their weight, indicating that the monkeys did consume their full ration (Fig. 5, linear regression pre-training weight - post-training weight 1: slope: 0.96, *p* =  1.7929e-05, R^2^ = 0.96; linear regression pre-training weight – post-training weight 2: slope: 0.86, *p* =  2.0648e-05, R^2^ = 0.96).

## 4 Discussion

The current set of experiments were conducted to explore refinements in our methods while training monkeys to acclimate to transport devices (t-boxes and primate chairs) voluntarily (i.e. without the use of pole and collar) for neuroscience experiments. Our experiments and analyses indicate that when training monkeys, if possible, working with them in pairs (Group 6 and 1 animal from Group 1) or in groups (Groups 3, 4 and 5) facilitated a reduction in the number of days spent acclimating to the transport devices. In addition, starting to train the monkeys as soon as they arrived in the primate unit helped to reduce the number of days (each day involved 1 x maximum of 30 min training session) involved in acclimation to their transport device. Furthermore, it was possible to train our monkeys to consistently enter either a t-box or a primate chair on a daily basis within a short duration (up to 10 days). Finally, when monkeys were required to enter a primate chair, it was also possible to consistently neck-plate them on a daily basis within 10 days. However, in both cases, PRT combined with some NRT techniques were required to ensure that all of the monkeys progressed with their training. Our training methods do not incorporate pole and collar techniques to transfer monkeys from their home enclosure to their transport device, as this is not allowed in the United Kingdom. The use of this combination of PRT and NRT in Groups 3, 4, 5 and 6, even without the use of pole and collar, decreased the overall number of days taken to acclimate monkeys to transport devices when compared to Groups 1 and 2 (singly-trained, or PRT only), thus reducing the time spent in this potentially stressful stage of training. The combined use of PRT and NRT but predominantly PRT, has also been reported to be effective during training primates for biomedical research (Graham et al., 2012; Graham, 2017; Wergård et al., 2015). For primate neuroscience research training, the majority of research labs in the US typically make use of PRT, or PRT and NRT in combination with the use of pole and collar techniques (Bliss-Moreau et al., 2013; McMillan et al., 2014, 2017). Thus together, these results and ours indicate that the use of predominantly PRT techniques combined with some NRT in the primate training program are essential. Our NRT techniques sometimes involved the use of the squeeze-back within the home enclosures (to encourage the monkey to come forward), or the use of the net (to encourage the monkey to move across into the ‘out-pen’) to ensure the monkey progressed in its training to acclimate to the transport devices. The use of the space reducers inside the chair, and or switching the monkey from the vertical to the horizontal position while inside the chair led to reliable and effective training of all of the monkeys when they had to be neck-plated to participate in the neuroscience experiments.

Previous evidence shows that monkeys and other primates will benefit from vicarious learning about the positive experiences of their fellow cagemates and from humans (Chang et al., 2011; Darby and Riopelle, 1959; Falcone et al., 2012a, 2012b; Fogassi et al., 2005; Meunier et al., 2007; Myers, 1970; Snowdon and Boe, 2003; Subiaul et al., 2007). Our results demonstrating that pair- or group-training reduced the number of sessions to acclimate to a transport device showed that the monkeys when being trained together facilitated the reduction in time taken. Opportunities for social interactions and social housing have been shown to be a beneficial husbandry practise for laboratory kept rhesus macaques as well (Hannibal et al., 2017). All of the monkeys involved in the current research were socially housed in pairs or groups of up to 8 monkeys.

We also kept our training sessions to acclimate to the transport devices short, i.e. for only up to 30 min per monkey per session, and we trained our monkeys for at least 5 consecutive sessions per week. Our data analyses of performance during these sessions showed that the desired behaviors were mainly exhibited by the monkeys in the first 10–20 min of the training sessions. Other researchers have also noted that short, frequent training sessions are most effective at promoting successful training of rhesus macaques (Fernström et al., 2009).

In addition, our analyses indicated that the use of PRT, combined with some NRT is desirable as this combination facilitated a reduction in the time spent training our monkeys. Our findings concur with other previously reported studies that have trained monkeys using PRT within a couple of weeks using either, pole and collar methods (Bliss-Moreau et al., 2013), or automated chair-training set-ups (Ponce et al., 2016). Interestingly in several rhesus macaque primate facilities, automated cage-based training methods have been successfully implemented, with these methods providing additional behavioral and cognitive enrichment (Calapai et al., 2017; Tulip et al., 2017). However, for the time being at least, in order to collect specific neuroscientific data (e.g. neurophysiological recordings, eye-tracking and neuroimaging), macaques are still required to enter transport devices from their home enclosures to allow these detailed recordings to be conducted. Our refinements in training monkeys to enter their transport devices from their home enclosure in a reduced number of sessions allowed them to progress in their training quickly so that they could engage with the neuroscience research sooner.

In our experience and as indicated in the data, the use of our PRT training techniques worked well for the majority of monkeys. However, critically, and as reported by others (Capitanio, 2017; Wergård et al., 2015), it was not possible to train all monkeys using PRT techniques alone. Therefore, we recommend a combination of PRT and NRT to support the effective and successful training of all monkeys to enter transport devices required for neuroscience experiments. However, we also advocate that the use of NRT techniques be considered after PRT techniques have been applied (Laule et al., 2003; Schapiro et al., 2003). That is, it must be emphasized that PRT methods should be used first and foremost to facilitate a positive relationship between the trainer and the monkey, as then the monkey is able to learn to associate receiving rewards from the trainer while interacting with its transport device and that entering its transport device is a rewarding experience. If NRT techniques are required, then they must be used for short, quick periods of time to be effective. The NRT technique allows the monkey to adapt its behavior in response to the quick presentation of the NRT reinforcer. As soon as the monkey has displayed a desired behavior be sure to remove the NRT reinforcer and provide the ‘bridge’ (secondary reinforcer) stimulus and/or offer them a highly palatable reward. Capitanio (2017) concludes that the need to resort to NRT for some monkeys to achieve the training goals is rooted in aspects of their individual temperament that may be indicated while they are infants.

As indicated for Group 1, who were trained individually, the majority of monkeys in the group acclimated relatively well to the t-box although it took them the most sessions. The one monkey that did not want to enter the t-box (M8, and was excluded from the statistical analyses) had to be paired with another cage-mate (M7) during its training sessions and eventually, through pairing for t-box entry, began to voluntarily enter its own t-box. The additional training requirements needed for this monkey still involved mainly PRT techniques, that is offering highly palatable food for interacting with the t-box (i.e. for desired behaviors) and the use of a bridging (secondary reinforcer) stimulus. However, some NRT techniques were also required, i.e. the use of the retractable rear walls in the home room enclosure to encourage the monkey to come forward towards the t-box, and showing the net. This monkey lived, as part of a group of three monkeys involved in immunology studies within our primate unit prior to joining our neuroscience research experiments. In our experience, some monkeys will do well in adapting to differences in training requirements and others will take a long time to re-train, or it may even be impossible given the time constraints of the program of research. Of the other two monkeys who were its cage-mates, one of the two was also able to be re-trained (as indicated above this monkey (M7) was used as a ‘teacher’ for this monkey), while another monkey (not included in any tables or statistics) in this group was not able to be re-trained at all and had to be used in an acute procedure.

Our experiments and analyses also highlighted the need to be able to utilize additional NRT techniques to facilitate training progress, e.g. the use of space reducers within the out-pens of the home enclosure and inside the primate chair. During the course of trialling our refinements in primate chair training, the primate chairs were modified for Groups 5 and 6 to incorporate adjustable side and back reducers to encourage the monkey to reposition itself when inside the primate chair for voluntary neck-plating. These NRT techniques must be removed immediately when the animal completes the desired behaviour to be effective, while the use of the ‘bridge’ stimulus and providing the monkey with a highly palatable reward must still be implemented for successful neck-plating.

In the neck-plate training, our analyses indicated that there was a transient behavioral regression for the monkeys upon advancing to the next training stage, that quickly recovered. For example, for Group 4 at Stage 3, adjusting to the chair moving from the vertical to horizontal (sphinx) position; and for Groups 5 and 6 at Session 4, the session immediately after having their neck plate closed, 62.5% of the monkeys (5/8) were not voluntarily presenting their head for neck plating. In these instances, and at other stages, if training is not progressing from the previous days, it is important for the trainer to be able to intervene quickly so that the monkey will still be able to succeed during its training session.

Sessions that involve regressions in behavioral training displayed by the monkey, can signify many things. Considering what the monkey had been experiencing in its previous training session is important to understand what the problem may be. When we were training our monkeys, we normally noticed these transient regressions in desired voluntary behaviors when the monkeys had experienced something unexpected (i.e. the regression after the first day of having the neck-plate closed). In these instances, it is important to maintain your boundaries and discipline; that is providing the highly palatable rewards for desired behaviors. In order to continue to do this in these training sessions though, the monkey trainer must create opportunities for the monkey to display a desired behavior to be able to reward it again so that they can rebuild their previously positive relationship with the monkey and allow it to move forward with its training. The use of the specific ‘bridge’ stimulus is also important at these times, as the monkey by now will have already been conditioned to understand that the use of this stimulus signals a desired behavior and that a reward is on the way soon. Two key aspects related to the use of the secondary reinforcer (‘bridge’) are important to emphasis here: firstly, the consistency of the nature of the ‘bridge’, meaning that if one decides to use a whistle, or sound, or a voice command then it must be used consistently rather than swapping between different stimuli. Secondly, one must be accurate in both the timing of the secondary reinforcer to signal the desired (rather than an unrelated) behavior and the pairing of the secondary reinforcer with the reward. The use of these PRT techniques help to re-establishing a positive relationship with the monkey.

Similar principles can also be applied throughout the training and testing sessions required for long-term neuroscience experiments as periods of transient regressions may occur at other time points as well (e.g. after surgeries or following breaks from testing). Some NRT techniques may also be necessary, but as previously indicated, if used, then they must be used quickly and removed immediately when the desired response is produced to be effective in encouraging the monkey to move forward in their training and progress in their experiments.

Finally, our data provide some timeframe guidelines that may be incorporated during training of monkeys for neuroscience experiments, when pole and collar methods are not used for training monkeys to enter transport devices. As our methods indicate, it is possible to train rhesus macaques to acclimate to transport devices necessary for daily neuroscience research experiments both quickly and effectively using mainly PRT, combined with some NRT techniques. In addition, beginning PRT training with the monkeys upon arrival in the unit, and pair- or group-training the monkeys helped to achieve a successfully acclimated monkey.

## Author’s contributions

SM, EP, PH and ASM designed the experiments. SM was the main primate trainer and also trained VP, AE and EP to work with the monkeys in our training procedures. VP (Group 5) and AE (Group 1) supported SM to train different groups of monkeys. All authors contributed to the preparation of the manuscript.

## Conflict of interest

The authors declare that they have no conflict of interest.

## Appendix A

Forage ingredients: a mixture of dried seeds and cereals, e.g. poppy, sunflower, and mustard seeds, uncooked rice, dried spilt green peas. Approximately 20 g per monkey, twice daily is distributed within the home cage across the substrate for them to forage for the seeds and cereals.

## Appendix B

### Mash ingredients

2–2.5 kg (2 scoops) of NHP mash powder

300 g Lilico Forage mix (1 scoop approx.)

400 g Raisins approx.

20 ml of Yumega

1400 ml of cold water

Mix up for at least 5 min in the mixer (we have an old bread bakery mixer for this). These ingredients will make up enough mash for approximately 40 monkeys as each monkey will received a specific weighed amount ranging between 120–220 g per day.
